# Can cardiac surgery be performed safely on patients with haematological malignancies

**DOI:** 10.5830/CVJA-2011-053

**Published:** 2012-05

**Authors:** A Guler, MA Sahin, F Cingoz, E Ozal, U Demirkilic, M Arslan

**Affiliations:** Department of Cardiovascular Surgery, Gulhane Military Medical Academy, Etlik, Ankara, Turkey; Department of Cardiovascular Surgery, Gulhane Military Medical Academy, Etlik, Ankara, Turkey; Department of Cardiovascular Surgery, Gulhane Military Medical Academy, Etlik, Ankara, Turkey; Department of Cardiovascular Surgery, Gulhane Military Medical Academy, Etlik, Ankara, Turkey; Department of Cardiovascular Surgery, Gulhane Military Medical Academy, Etlik, Ankara, Turkey; Department of Cardiovascular Surgery, Gulhane Military Medical Academy, Etlik, Ankara, Turkey

**Keywords:** ematological malignancy, cardiac surgery, intracranial bleeding

## Abstract

**Introduction:**

Surgical strategy in patients with haematological malignancies must be planned and carried out with the specific aim of decreasing postoperative complications. The aim of this study was to present our experience on patients previously diagnosed with haematological malignancies who subsequently underwent cardiac surgery. We include data to assist other surgeons predict factors affecting postoperative morbidity and mortality in this group of patients.

**Methods:**

Fifteen patients diagnosed with haematological malignancies who had cardiac surgery were retrospectively analysed. Eight patients had chronic lymphocytic leukaemia, six had non-Hodgkin’s lymphoma and the rest had chronic myelocytic leukaemia. Coronary artery bypass graft surgery was performed on all of them.

**Results:**

There were no hospital mortalities. The average follow-up period was 35 ± 11 (23–56) months. Three patients required early postoperative re-operation because of excessive bleeding. No mortalities were seen in the early postoperative period. There were five (33%) deaths during the late follow-up period. Three patients were lost due to intracranial bleeding (confirmed by autopsy) in the 16th, 23rd and 38th months after surgery. The remaining two patients had sudden death in the eighth and 55th months from non-detectable causes.

**Conclusion:**

Conclusion: Cardiac surgery can be performed with acceptable early postoperative outcomes in patients with haematological malignancies. Intracranial bleeding is an important factor contributing to late mortality and patient selection and risk stratification are crucial to improving surgical benefits.

## Abstract

Increased surgical experience and technological advances in cardiac surgery have encouraged surgeons to perform complex cardiac operations in patients with unrelated complications. Several procedures are performed nowadays on patients with co-morbidity factors, with acceptable morbidity and mortality rates.^1^

Haematological malignancies are diagnosed in all age groups but the chronic forms are predominantly seen in elderly populations.^2^ Great strides have been made to improve the quality of life of these patients and many clinicians are now focusing on finding solutions to other symptoms these patients may have. In an era when atherosclerotic heart disease shows an increasing prevalence, cardiac surgeons are encountering this population more frequently.

The operative risk of patients with malignant haematological disorders is increased, as this may include coagulation defects, changes in blood viscosity, immune suppression and bone marrow insufficiency.^3^ When surgically treating these patients, one must be concerned about postoperative infection, haemorrhage and leukaemic transformation. Surgical trauma and cardiopulmonary bypass (CPB), because of their immune-depressing effects, have the potential risk of increasing the haematological problems, leading to fatal or morbid complications.^4^

There are few reports on how to deal with these patients, and also little knowledge on their progress after cardiac surgery. Due to these concerns, the aim of our study was to detail our clinical experience and data on the postoperative period.

## Methods

We retrospectively reviewed hospital records of 15 patients with haematological malignancies who underwent cardiac surgery at the Cardiovascular Surgery Department of our institution between 2003 and 2009. Eight patients suffered from chronic lymphocytic leukemia (CLL), six had non-Hodgkin’s lymphoma (NHL), and the rest had chronic myelocytic leukaemia. Coronary artery bypass graft (CABG) surgery was performed on all patients.

The diagnosis of haematological malignancy was assigned based on the international ICD-10 code. Patients received routine intravenous antibiotic prophylaxis (cephazolin Na 1 g) for three days, beginning the night before surgery. CPB was performed after the standard heparinisation (ACT > 400 s). All the patients were given antegrade cold crystalloid cardioplegia with moderate hypothermia, and topical cold slush solution was used.

The left internal mammary artery (LIMA) and saphenous vein grafts were prepared for CABG surgery. Distal anastomoses were performed with 7/0 polypropylene sutures and a continuous suture technique. Proximal anastomoses were performed with 6/0 polypropylene sutures and a continuous suture technique with a side clamp. Salicylic acid 100 mg was started on the first postoperative day in all patients.

Patients’ hospital charts, demographics, peri-operative data and complications were reviewed. Follow-up data were obtained by review of subsequent hospital admissions and telephone interviews. The average follow-up period was 35 ± 11 (23–56) months.

In statistical analysis, values were expressed as mean ± standard deviation for continuous variables, and number and percentage for dichotomous variables. In comparisons, the Student’s *t*-test was used for continuous variables and the Mann-Whitney *U*-test for dichotomous variables. Comparison of gender was done using the Chi-square test. A *p*-value below 0.05 was set as significant.

## Results

There were a total of 15 patients, 11 were male and four female. Their pre-operative data are summarised in Table 1. Mean age was 65 ± 14 (range 27–76) years. The mean time interval from the diagnosis of the haematological malignancy to the CABG surgery was 4.6 years (7 months to 9 years). All were in remission and under supervision of the Haematology Department.

**Table 1:** Clinical Data

*Diagnosis*	*CLL*	*Non-Hodkgin’s lymphoma*	p-*value*	*CML*
Number	8	6		1
Gender (male/female)	6/2	4/2 0.733	0.733**	M
Age (years)	66 ± 16	60 ± 12	0.128*	78
Body mass index	24 ± 4.2	26.5 ± 4.9	0.096*	22
Pre-operative EF (%)	49 ± 13	52 ± 18	0.226*	44
Pre-operative haemoglobin (g/dl)	13.3 ± 3.6	12.8 ± 2.9	0.258*	15.4
Post-operative haemoglobin (g/dl)	11.9 ± 7.2	10.8 ± 8.6	0.086*	12.3
Pre-operative platelets (× 109 /dl)	206 ± 74	254 ± 89	0.068*	139
Post-operative platelets (× 109 /dl)	174 ± 51	152 ± 73	0.070*	128
Pre-operative prothrombin time (s)	12.8 ± 4.1	12.5 ± 2.8	0.825*	13.3
Post-operative prothrombin time (s)	13.2 ± 7.2	12.9 ± 9.4	0.652*	13.1
Pre-operative WBC (× 109 /dl)	33.2 ± 9.1	38.5 ± 10.8	0.075*	20.0
Post-operative WBC (× 109 /dl)	36.1 ± 1.4	34.8 ± 5.3	0.348*	17

CLL: chronic lymphocytic leukemia, CML: chronic myelocytic leukemia, EF: ejection fraction, WBC: white blood cells, Postoperative: 14th day. *Student’s *t*-test, **Chi-square test; *p*-value represents the comparison between CLL and non-Hodkgin’s lymphoma patients.

An average of 2.75 ± 1.2 grafts was placed in the 15 patients undergoing isolated CABG. LIMA grafts were used in 14 patients. All were operated on with an arrested heart under CPB. Two patients required intra-aortic balloon pump during the peri-operative period. Haematology reports reflected the expected ranges in all patients during the postoperative period.

In patients with CLL and NHL, the average length of stay in ICU was 1.2 ± 0.3 and 1.5 ± 0.5 days, respectively. The average of packed red blood cells required was 2.1 ± 0.6 and 1.5 ± 0.6 units, respectively, and the average of fresh frozen plasma required was 1.3 ± 0.5 and 1.5 ± 0.6 units, respectively. There was no apparent difference in the postoperative course and mean postoperative stay between the two groups (Table 2).

**Table 2:** Peri-Operative Data

*Diagnosis*	*CLL*	*Non-Hodkgin’s lymphoma*	p-*value*	*CML*
CPB time (min)	83 ± 21	88 ± 16	0.215*	95
Cross-clamp time (min)	64 ± 19	69 ± 24	0.083*	72
Packed red blood cells (units)	2.1 ± 0.6	2.3 ± 0.8	0.573**	3
Fresh frozen plasma (units)	1.3 ± 0.5	1.5 ± 0.6	0.755**	1
Post-operative IABP	1	1	0.950**	–
ICU stay (days)	1.2 ± 0.3	1.5 ± 0.5	0.491*	1
Mortality early/late	–/3	–/2	0.950**	–
Bleeding complications	–	3	0.030**	
Infection	1	–	0.755**	

CLL: chronic lymphocytic leukaemia, CML: chronic myelocytic leukaemia, CPB: cardiopulmonary bypass, IABP: intra-aortic balloon pump, ICU: intensive care unit, *Student’s *t*-test, **Mann-Whitney *U*-test, *p*-value represents the comparison between CLL and non-Hodkgin’s lymphoma patients.

Four postoperative complications occurred in four patients (26%). Three of them required early re-operation because of bleeding. They were respectively, 55-, 63- and 69-year-old male patients, diagnosed with NHL. More bleeding was seen in the patients with NHL than in those with CLL, and the difference was statistically significant (*p* < 0.05). The hospital stay was uneventful after the second operation.

A 27-year-old male patient diagnosed with CLL was hospitalised one month after the first operation due to mediastinitis. This patient was treated with antibiotics according to bacterial culture and he then underwent sternal dehiscence revision surgery. The hospital course was uneventful after the second operation and he soon returned to work. Clinical and peri-operative variables of patients with CLL and NHL were similar (Tables 1, 2). No mortality was seen in the early postoperative period.

The three-year survival rate was 80%. There were five (33%) late deaths during the follow-up period. All deaths were of a non-cardiac nature. Three patients were lost due to intracranial bleeding in the 16th, 23rd and 38th months after surgery. The other two patients had sudden death in the eighth and 55th months and the reason for death could not be determined. One of these patients was brought to the emergency department with cardiopulmonary arrest. Resuscitation was performed but the patient died and the family refused an autopsy.

## Discussion

Haematological malignancies, particularly the lymphocytic types, affect mainly elderly patients. Survival of these patients can range from one year to several decades.^2^,^5^ Over the past few years, treatment options have continued to improve survival rates. Cardiac surgical experience in patients with haematological malignancies is limited and detailed investigation is mandatory in decision making.^2^

It is obvious that CPB affects all systems, including the haematopoietic system. CPB, which aggravates cell damage, also has immune-depressant properties, resulting in an increased incidence of infection.^6^,^7^ Furthermore, haematological malignancies may lead to antibody deficiency, leucopenia or impaired T-cell function. Previous reports demonstrate infection as the primary cause of morbidity.^2^,^3^,^8^ Some studies show morbidity rates of between 23 and 57%. Samuels *et al*. reported the incidence of infectious complications as 42%, underlining the major role they play in the hospital stay.^2^,^5^ Some investigations emphasise the use of additional intravenous immunoglobulin, as broad-spectrum antibiotic prophylaxis was found to be insufficient to prevent or control infection in these patients.^2^

According to the advice of our infections committee, we used only cephazolin Na for prophylactic antibiotic therapy. Mediastinitis was observed in one patient and it was the only infection-related complication encountered in our cases. This was an acceptable infection rate and comparable with that of open-heart surgery in the general population.

White blood cell count generally increases after open-heart surgery, and CPB may stimulate a leucomoid reaction, which can lead to a relapse in an otherwise quiescent illness.^6^,^7^ In our study, white blood cell count increased between acceptable ranges, as seen in Table 1. The general opinion on cardiac surgery is that it does not exacerbate haematological malignancies in low-risk patients or those with low-grade disease (in the remission period). However patients with an intermediate to high risk or grade of disease may show progression and this may be the cause of late death.^5^ Our patients were all in complete remission and there was no leucomoid reaction or relapse of the disease in the follow-up period, as confirmed by the Haematology Department.

Bleeding is another potential complication in this group of patients. Fecher *et al*.^5^ reported a 16.6% rate of bleeding and it was the main postoperative complication they observed. They had only one mediastinal bleeding that led to re-operation, and two cases of gastrointestinal and one of femoral artery haemorrhage. They were not group related.

In our series, three patients (20%) had excessive bleeding in the early postoperative period, showing a similar rate to that of Fecher *et al*.^5^ However, all were of mediastinal origin and led to re-operation and all were patients with NHL. One of the cases of haemorrhage was surgery related while a general leakage was observed in the other two cases. We did not detect any significantly low platelet count or elevated INR peri-operatively.

Bleeding was the only statistically significant difference observed between the patients with CLL and NHL. We did not find any reports in the literature regarding NHL and tendency to haemorrhage and we felt that statistical analysis was not feasible for some factors because of the small size of our study group. However, it may be valuable in future clinical decisions.

This article has some limitations. It was retrospective and lacked a control group of standard patients with CABG. The number of patients and types of malignancies were different among different institutions, therefore it was difficult to find a large series of patients to determine the morbidity and mortality rates in a homogeneous group.

Incomplete follow-up data was another shortcoming. In previous reports, 30-day mortality rates ranged from zero to 17%.^2^,^5^,^9^ In our series, there was no early mortality, but late mortality was somewhat higher (33%). Three patients died because of intracranial bleeding and two others due to unknown reasons.

Samuels *et al*. pointed out that these patients’ long-term outcomes are variable and non-cardiac related.^2^ The high late-mortality rate of our patients may be partially related to our limited experience in this specific group of patients. Appropriate long-term anti-aggregant therapy should be designed in collaboration with haematology departments.

We surmise that the small number of patients seriously affected the late-period outcomes and no predictive factors for identifying such high-risk patients were found. Although intracranial bleeding was a significant mortality factor, prothrombin time, bleeding time and thrombocyte level were in the normal range in all patients. Aspirin 100 mg was the anti-aggregant therapy and it may have been responsible for intracranial bleeding. However, there is no evidence and future guidelines should describe a detailed approach and treatment.

It is reasonable to use less-invasive techniques (percutaneous coronary interventions) in high-risk patients when they meet the operative indications. However, these procedures require heavy post-intervention anti-agregant therapy, and the risk for intracranial bleeding would therefore remain high.

Predictive scores such as the Euroscore or the Society of Thoracic Surgeons’ score have no predictive values for mortality and morbidity in patients with haematological malignancies undergoing cardiac surgery. Therefore, future studies giving accurate rates of post-operative morbidity or mortality would be of great use to provide a general approach for these patients.

## Conclusion

Cardiac surgery can be undergone with acceptable early postoperative outcomes in patients with haematological malignancies. The expectations of the patient and surgeon should be appropriately discussed and the medical team should be focused on potential complications such as bleeding, infection and prolonged hospital stay. Long-term multidisciplinary follow up and adequate medical treatment are essential in prolonging survival.
